# Stereotactic ablative brachytherapy versus percutaneous microwave ablation as salvage treatments for lung oligometastasis from colorectal cancer

**DOI:** 10.1186/s12885-024-12163-3

**Published:** 2024-04-16

**Authors:** Yuliang Li, Zitong Chen, Shuhui Tian, Xujian Han, Changjun Wang, Yongzheng Wang, Bin Liu

**Affiliations:** 1 Department of Interventional Medicine and Minimally Invasive Oncology, The Second Hospital of Shandong University, No. 247 Beiyuan Street, Jinan, 250033 PR of China; 2 Interventional Oncology Institute, Shandong University, Jinan, PR of China; 3 Department of Radiology, Shandong Provincial Hospital, Jinan, PR of China; 4 Department of Radiology, People’s Hospital of Jiyang County, Jinan, PR of China

**Keywords:** Stereotactic ablative brachytherapy, Microwave ablation, Oligometastasis, Lung, Colorectal cancer

## Abstract

**Background:**

The treatment for lung oligometastasis from colorectal cancer (CRC) remains challenging. This retrospective study aimed to compare the local tumor control, survival and procedure-related complications in CRC patients undergoing low-dose rate stereotactic ablative brachytherapy (L-SABT) versus percutaneous microwave ablation (MWA) for lung oligometastasis.

**Methods:**

Patients between November 2017 and December 2020 were retrospectively analyzed. Local tumor progression-free survival (LTPFS) and overall survival (OS) were analyzed in the entire cohort as well as by stratified analysis based on the minimal ablation margin (MAM) around the tumor.

**Results:**

The final analysis included 122 patients: 74 and 48 in the brachytherapy and MWA groups, respectively, with a median follow-up of 30.5 and 35.3 months. The 1- and 3-year LTPFS rate was 54.1% and 40.5% in the brachytherapy group versus 58.3% and 41.7% in the MWA group (*P* = 0.524 and 0.889, respectively). The 1- and 3-year OS rate was 75.7% and 48.6% versus 75.0% and 50.0% (*P* = 0.775 and 0.918, respectively). Neither LTPFS nor OS differed significantly between the patients with MAM of 5–10 mm versus > 10 mm. Pulmonary complication rate did not differ in the overall analysis, but was significantly higher in the MWA group in the subgroup analysis that only included patients with lesion within 10 mm from the key structures (*P* = 0.005). The increased complications was primarily bronchopleural fistula.

**Conclusions:**

Considering the caveats associated with radioisotope use in L-SABT, MWA is generally preferable. In patients with lesion within 10 mm from the key pulmonary structures, however, L-SABT could be considered as an alternative due to lower risk of bronchopleural fistula.

## Introduction

Metastasis to the lungs occurs in 10–15% of the patients with colorectal cancer (CRC) [1]. Local control of the metastatic lesions in the lungs is associated with improved survival [2], and particularly in patients who could not tolerate chemotherapy [3]. Lung metastasectomy in CRC patients with oligometastasis to the lungs could improve patients’ prognosis, with 5-year overall survival (OS) rate of 53.5% [4]. However, the majority of the patients are not appropriate candidate for lung metastasectomy [5].

Local treatments for such patients include thermal ablation [6–8] and low-dose rate stereotactic ablative brachytherapy (L-SABT) [9, 10]. Due to distinct physical properties, L-SABT and thermal ablation have different advantages in the treatment of CRC lung oligometastasis. To our best knowledge, no studies that compared L-SABT versus percutaneous microwave ablation (MWA) in CRC patients with oligometastasis to the lungs was reported. Therefore, we conducted a retrospective analysis to compare local tumor control, survival and procedure-related complications in patients undergoing L-SABT versus MWA in such patients.

## Materials and methods

### Patient population

In this multicenter retrospective analysis, we screened all CRC patients treated with either L-SABT or percutaneous MWA for oligometastasis to the lungs at authors’ centers during a period from November 2017 and December 2020. Each participant provided written informed consent, and all methods were performed in accordance with approved guidelines and the Declaration of Helsinki. The study was approved by the institutional review board of the Second Hospital of Shandong University [KYLL-2021(KJ)P-0363].

All the primary tumors had been treated with radical surgery, and the diagnosis of CRC was based on pathology. Oligometastasis to the lungs was established based on the presence of no more than three lesions on contrast-enhanced CT (CECT) and absence of metastasis to sites other than the lungs [11]. For patients in the final analysis, all the following criteria must be met: (1) ≤ 3 metastatic lesions in the lungs; (2) after standard platinum-based chemotherapy and docetaxel-based chemotherapy; (3) no prior local treatment to lung metastasis; (4) patients deemed medically unsuitable for surgery or had refused surgery and external beam radiotherapy; (5) Eastern Cooperative Oncology Group performance status 0 or1.

Patients with one of more of the following conditions were excluded from the final analysis: (1) active primary tumor and extrathoracic disease; (2) severe cardiac insufficiency (New York Heart Association class III or IV), advanced lung diseases determined by consultation with respiratory disease specialists, poor liver reserve (Child–Pugh class C), or severe renal dysfunction (stage 3 or higher chronic kidney disease); (3) severe coagulopathy (international standardized ratio > 2.0 and/or platelet count ≤ 60 × 10^9^/L).

CECT was used for pretreatment evaluation. Lab panel including standard blood count, coagulation function, liver function, and carcinoembryonic antigen (CEA) tests were also performed.

### Intervention

In patients with target lesions confined to one lung only, the treatment (L-SABT or MWA) was completed in one session. In patients with target lesions involving both lungs, treatment was conducted in two sessions separated by at least 3 weeks.

#### L-SABT

**Fig. 1:**
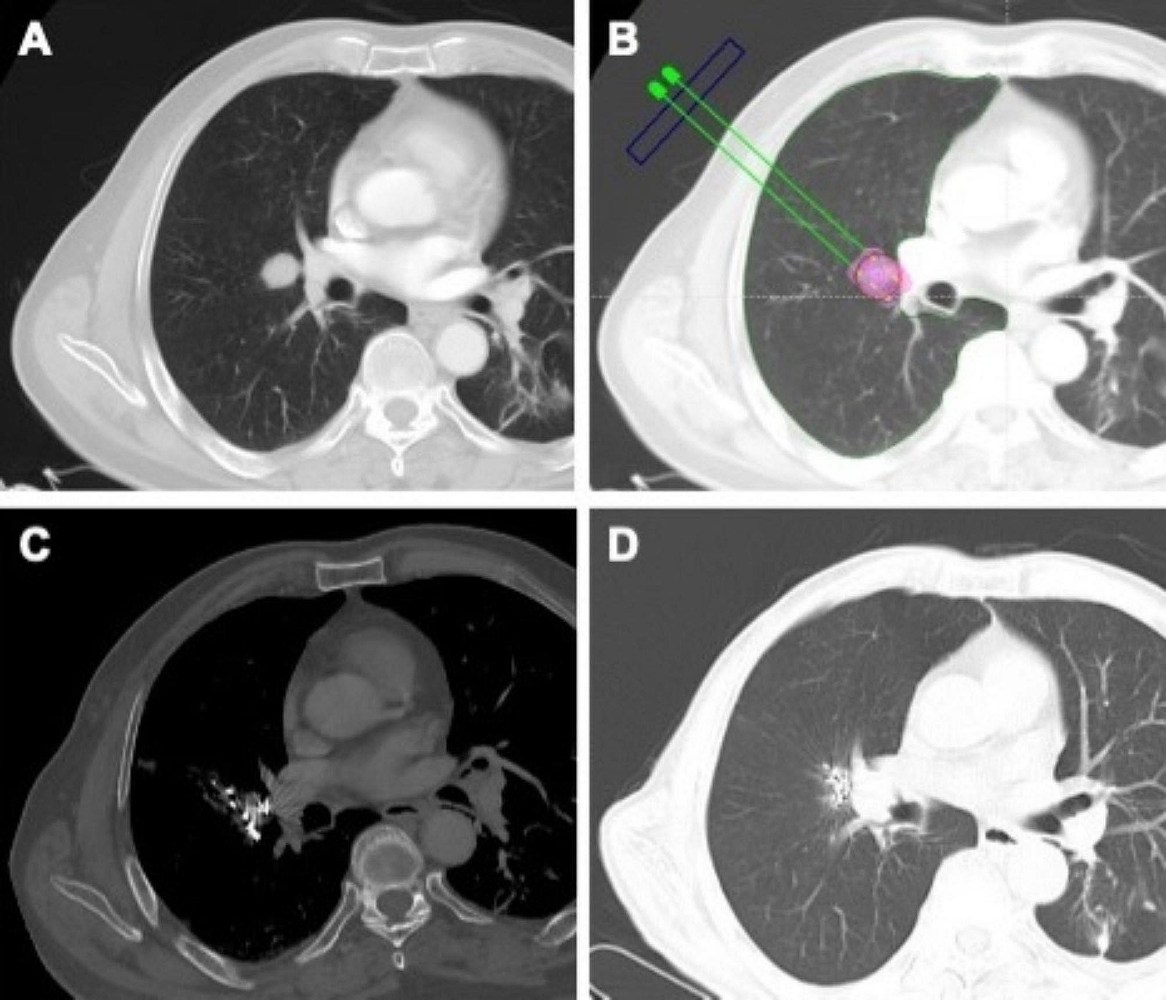
A representative case of brachytherapy. **A** A metastatic lesion adjacent to the right hilum (yellow arrow). **B** Treatment plan before brachytherapy (red region—D90 coverage area; green line—delineated organ at risk). **C** CT scan after brachytherapy (high-density dots—implanted iodine-125 seeds). **D** Follow-up CT at 16 months

Pretreatment plan was conducted using the treatment planning system (TPS) (Fig. 1A-B). The gross target volume (GTV) and organs at risk were delineated according to CECT [12, 13]. The planning target volume covered one more centimeter beyond the margin of GTV. In patients with atelectasis, the target volume was delineated using magnetic resonance imaging or ^18^F-fluorodeoxyglucose positron emission tomography (PET-CT) [13].

**Fig. 2:**
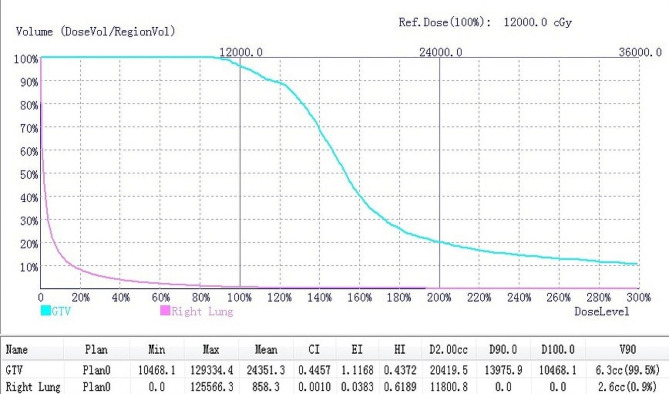
Dose verification after brachytherapy

The procedure was performed under moderate sedation with a 0.1-mg fentanyl bolus and dexmedetomidine infusion at a rate of 1.5 µg/kg/h. The puncture plane (intercostal space) was selected based on tumor location and size. Bone drilling or artificial pneumothorax was conducted if the bone hampered the puncturing [9, 13]. Iodine-125 seeds were implanted via an 18-gauge puncture needle according to the TPS plan (Fig. 1C). Fluorouracil with a 0.05-ml bolus for one seed was injected through the puncture needle to prevent tumor seeding. Upon procedure completion, chest CT scan was conducted to verify the distribution of iodine-125 seeds (Fig. 1D). Dosage was verified to confirm whether it was accord with the pretreatment TPS plan (Fig. 2).

#### MWA

**Fig. 3:**
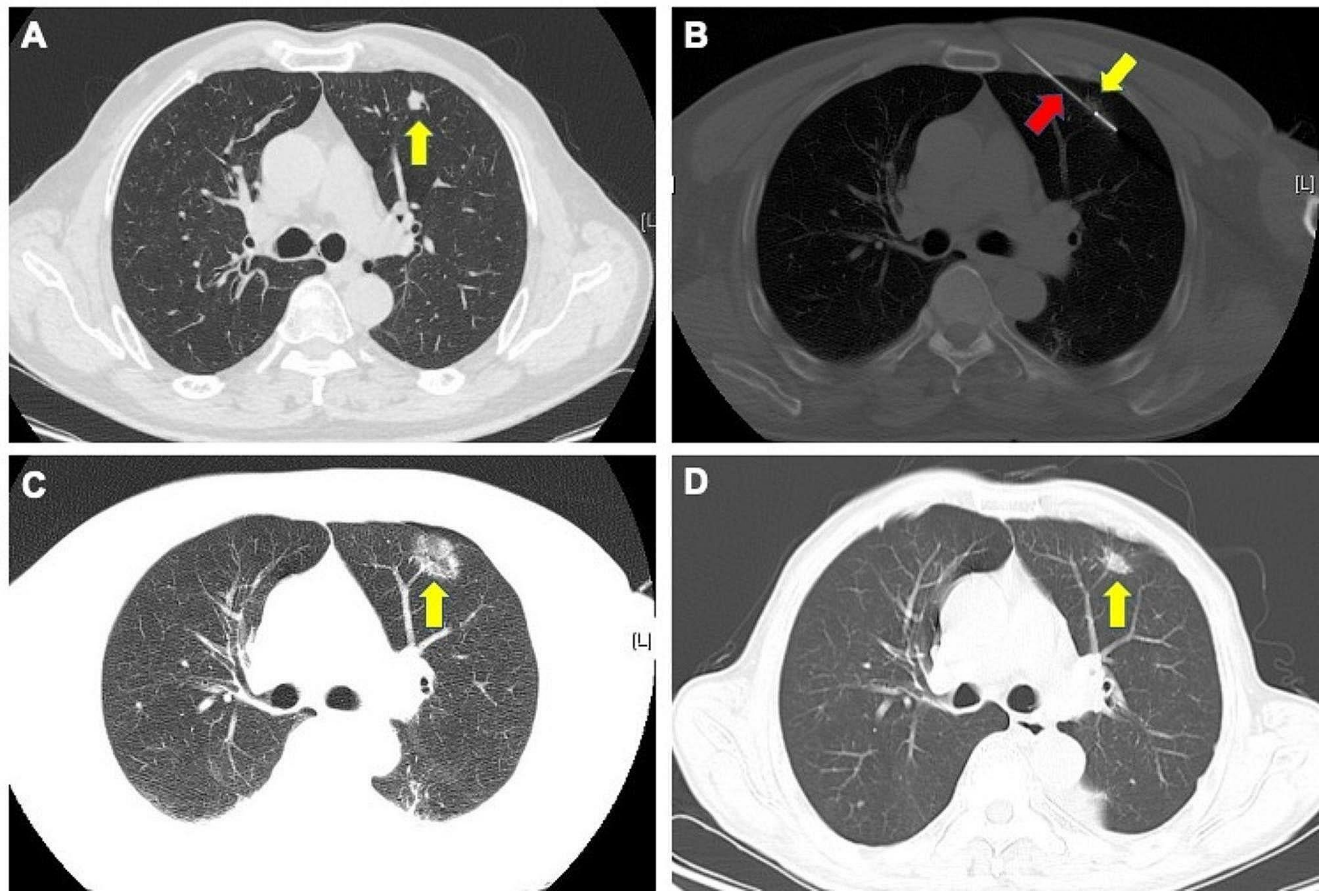
A representative case of MWA. **A** A metastatic lesion located in the left lung (yellow arrow). **B** Punctured applicator (red arrow) through the center of metastasis (yellow arrow). **C** A ground-glass opacity (yellow arrow) indicating complete ablation after the procedure. **D** Follow-up CT at 12 months

The procedure was performed under moderate sedation identical to that used for L-SABT. The treatment plan was selected based on the tumor location and size, and to avoid the bones, large vessels, and pulmonary fissures (Fig. 3A). The applicator (ECO-100AL6, Φ1.6; Nanjing, China) was inserted into the lesion under CT guidance (Fig. 3B). The ablation power and time were selected based on the size and geometry of the lesions. Minimal ablation margin (MAM) was measured according to Kurilova’s study [14], which shoule be at least 5 mm beyond the pre-procedure tumor borders (Fig. 3C) [15, 16].

### Follow-up

Follow-up was conducted at 1 and 3 months and every 3 months thereafter [16], and consisted a complete physical examination, standard lab panel that included CEA testing, CECT covering chest-abdomen-pelvis, and dose verification by TPS (for the brachytherapy group only).

### Outcome

**Fig. 4:**
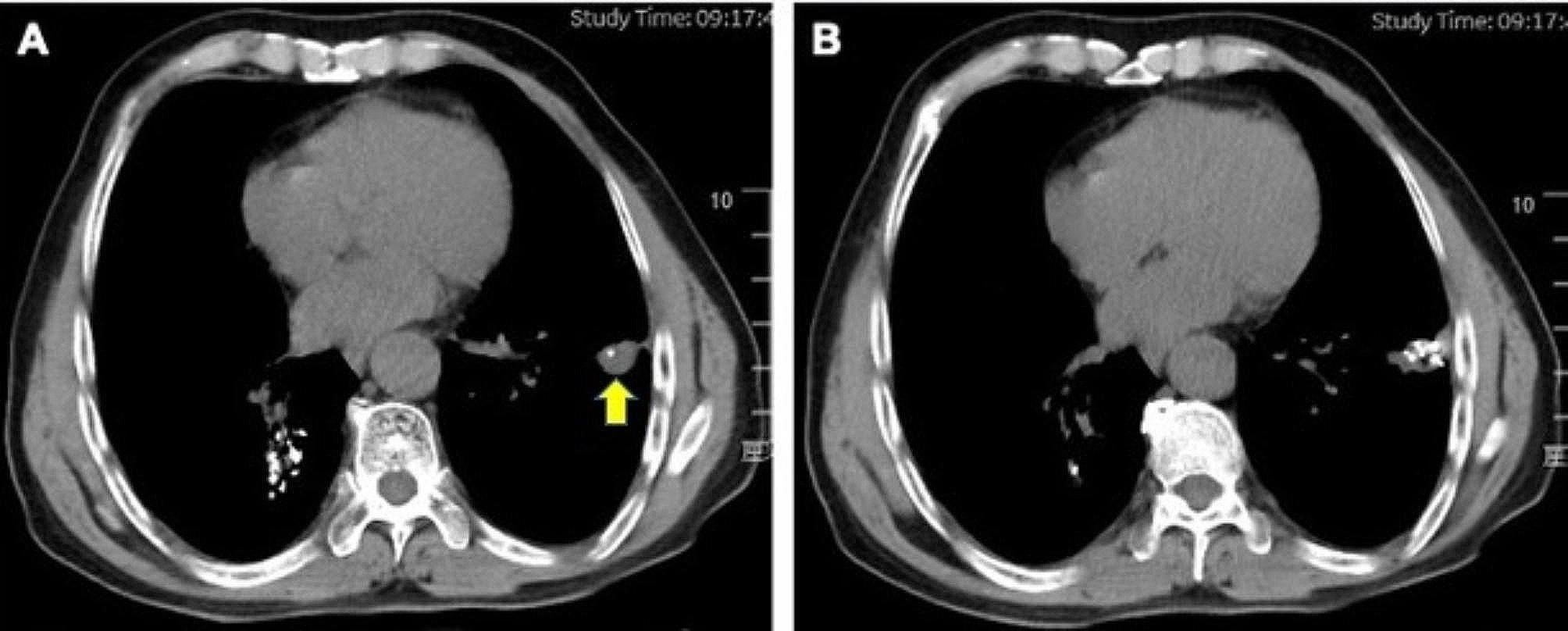
Another representative case of L-SABT. Follow-up CT at 5 months indicated local tumor progression (yellow arrow)

The outcome of primary interest was local tumor progression-free survival (LTPFS), defined as the duration from the treatment (L-SABT or MWA) to local tumor progression (LTP, Fig. 4) or the last follow-up date. OS was defined as the duration from the treatment to death or the last follow-up date. LTP was defined as the evidence of new lesions within 1 cm from the ablation zone seen on CECT [17].

Treatment response was evaluated using the Response Evaluation Criteria in Solid Tumors (RECIST) version 1.1 [18]. Complications were recorded based on the Common Terminology Criteria for Adverse Events v5.0, and were graded according to the Clavien-Dindo classification [19]. Bronchopleural fistula was defined as a leakage of inspired air from the airways into the pleural space for more than 24 h despite of treatment [20].

### Statistical analysis

Continuous variables with normal distribution were presented as mean and standard deviation SD, and analyzed using Student’s t-test for independent samples. Continuous variables with skewed distribution were presented as median and interquartile range (IQR), and analyzed using the Mann–Whitney U test. Categorical variables were presented as number and percentage, and analyzed using χ^2^ or Fisher exact test, as appropriate. Pulmonary complications were analyzed in the overall cohort as well as in subgroup analyses stratified based on distance of the lesion to key structures (≤ 10 vs. > 10 mm from the hilum, pleura or interlobar fissure).

Multivariable Cox proportional hazards regression was conducted to identify factors associated with LTPFS and OS. Age and CEA were considered as a categorical variable in the regression (cutoff at 65 years and 10 ng/ml, respectively) based on previous studies [14, 21]. Factors with *P* < 0.1 in the univariable regression were entered into the multivariate analysis as independent variables. Results of the regression are shown as hazard ratio (HR) and 95% confidence interval (CI). *P* < 0.05 (2-sided) was considered statistical significance. All statistical analyses were performed using SPSS 24.0 (IBM Inc., Armonk, NY, USA).

## Results

### Patient and procedural characteristics

The final analysis included 122 patients: 74 in the brachytherapy group and 48 in the MWA group. The two groups did not differ significantly in sex, age, primary tumor location, historical characteristics, tumor number, distribution of lung metastasis, metachronous or synchronous lung metastasis, CEA level and administration of TKI and/or immunotherapy (Table 1).

**Table 1:** Demographic and baseline characteristics of the entire cohort

Characteristics	Brachytherapy group (*N* = 74)	MWA group (*N* = 48)	P value
Sex, no. (%) Male Female	42 (56.8%) 32 (43.2%)	26 (54.2%) 22 (45.8%)	0.842
Mean age (years) Age (years), no. (%) <65 ≥65 Primary tumor location, no. (%) Right colon Left colon Rectum Historical characteristics, no. (%) Adenocarcinoma Others Maximum tumor diameter (cm, mean ± SD) Tumor size, no. (%) ≤3 cm >3 cm Tumor number, no. (%) 1 2 3 Distribution of lung metastasis, no. (%) Unilateral Bilateral	63.6 ± 8.7 46 (62.2%) 28 (37.8%) 30 (40.5%) 20 (27%) 22 (32.5) 67 (90.5%) 7 (9.5%) 5.7 ± 2.4 38 (51.4%) 36 (48.6%) 18(24.3%) 34(46%) 22(29.7%) 44 (59.5%) 30 (40.5%)	58.5 ± 9.2 28 (58.3%) 20 (41.7%) 18 (37.5%) 16 (33.3%) 14 (29.2%) 43 (89.6%) 5 (10.4%) 2.5 ± 0.8 40 (83.3%) 8 (16.7%) 18(37.5%) 14(29.2%) 16(33.3%) 24 (50%) 24 (50%)	0.357 0.765 0.869 > 0.99 0.027 0.011 0.376 0.467
Location of lung metastasis, no. (%) Adjacent to the hilum Adjacent to the pleura/interlobar fissure	28 (37.8%) 26 (35.1%)	6 (12.5%) 12 (25%)	0.016
None of the above Lung metastasis, no. (%) Metachronous Synchronous CEA (ng/ml), no. (%) <10 ≥10 Administration of TKI and/or immunotherapy, no. (%) Yes No	20 (27.1%) 56 (75.7%) 18 (24.3%) 28 (37.8%) 46 (62.2%) 24 (32.4%) 50 (67.6%)	30 (62.5%) 34 (70.8%) 14 (29.2%) 24 (50%) 24 (50%) 14 (29.2%) 34 (70.8%)	0.674 0.348 0.842

MWA, microwave ablation; carcinoembryonic antigen, CEA.

The maximum tumor diameter was 5.7 ± 2.4 cm in the brachytherapy group versus 2.5 ± 0.8 cm in the MWA group (*P* = 0.027). The percentage of patients with lesions within 10 mm from the hilum, pleura or interlobar fissure was 73% (54/74) in the brachytherapy group versus 37.5% (18/48) in the MWA group (*P* = 0.016) (Table 1).

The median prescription dose in the brachytherapy group was 120 Gy (IQR: 110, 125); the median activity of iodine-125 seeds was 0.6 mCi (IQR: 0.54–0.68). Bone drilling was required in 15 patients (20.3%). The median number of seeds was 55 (IQR: 30, 75), and the median number of needles was 8 (IQR: 6, 11). The mean D90 was 138.5 ± 17.2 Gy.

The procedure time was 57 ± 13 min in the brachytherapy group versus 31 ± 7 min in the MWA group (*P* = 0.036) (Table 2). There were no significant differences regarding CT fluoroscopy time and radiation dosage between the two groups.

**Table 2:** Outcome analysis of the entire cohort

Characteristics	Brachytherapy group (*N* = 74)	MWA group (*N* = 48)	P value
Procedure time (minutes) MAM (mm)	57 ± 13 -	31 ± 7 8.8 ± 2.1	0.036 -
LTPFS at 1 year, no. (%) Yes No	40 (54.1%) 34 (45.9%)	28 (58.3%) 20 (41.7%)	0.524
LTPFS at 3 years, no. (%) Yes No Median LTPFS (months) OS at 1 year, no. (%) Yes No OS at 3 years, no. (%) Yes No	30 (40.5%) 44 (59.5%) 17.3 (95% CI: 8.4–19.2) 56 (75.7%) 18 (24.3%) 36 (48.6%) 38 (51.4%)	20 (41.7%) 28 (58.3%) 19.8 (95% CI: 8.3–21.9) 36 (75%) 12 (25%) 24 (50%) 24 (50%)	0.889 0.871 0.775 0.918
Median OS (months) Complications, no. (%) Pneumothorax Grade 1 Grade 2 Bronchopleural fistula Grade 3 Pleural effusion Grade 1 Grade 2 Hydropneumothorax Grade 1 Grade 2 Fever Grade 1 Grade 2 Total, no. (%)	21.6 (95% CI: 15.3–33.9) 18 (24.3%) 2 (2.7%) 0 6 (8.1%) 0 6 (8.1%) 2 (2.7%) 0 0 34 (45.9%)	23.4 (95% CI: 15.4–36.7) 14 (29.2%) 2 (4.2%) 6 (12.5%) 2 (4.2%) 0 2 (4.2%) 0 6 (12.5%) 0 32 (66.7%)	0.865 0.113

MAM, minimal ablation margin; MWA, microwave ablation; LTPFS, local tumor progression-free survival; OS, overall survival.

The mean MAM around the tumor in the MWA group was 8.8 ± 2.1 mm. The technical success rate was 100% in both groups. The median follow-up time was 30.5 months (95% confidence interval [CI]: 16.3–44.6) in the brachytherapy group versus 35.3 months (95% CI: 19.6–51.7) in the MWA group (*P* = 0.38).

### Outcome

#### LTPFS

**Fig. 5:**
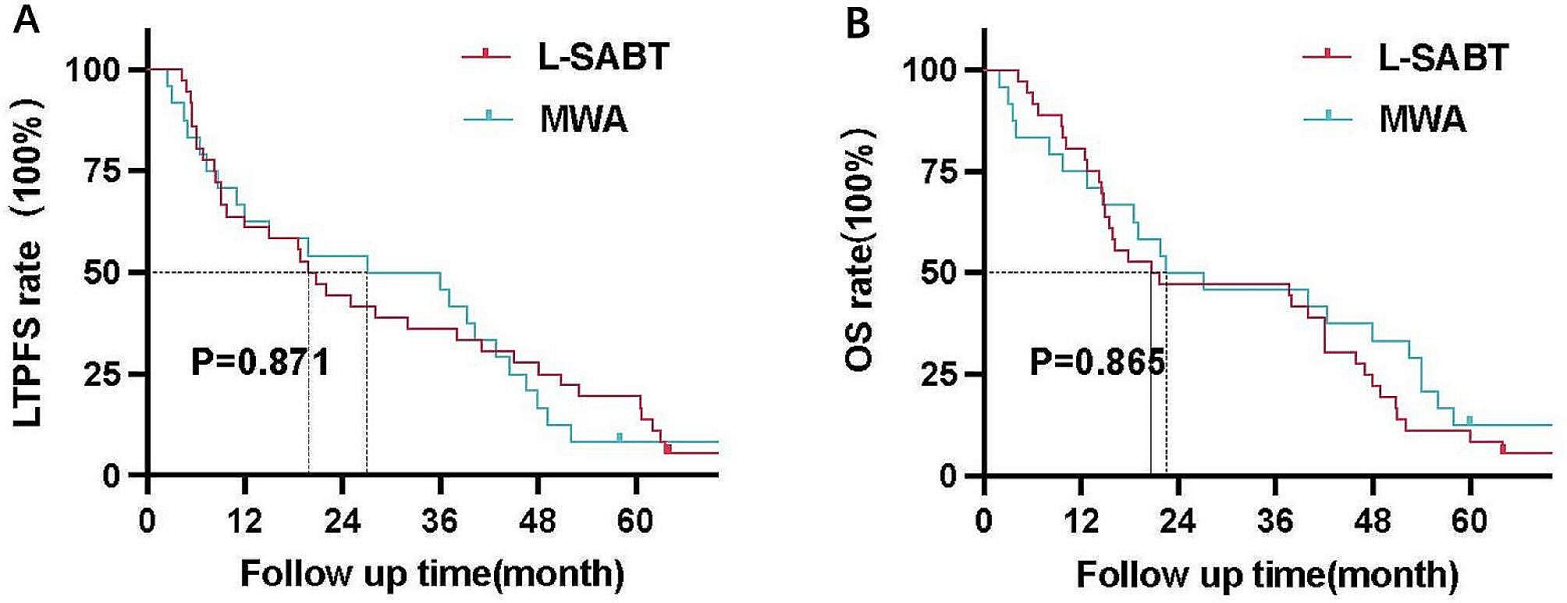
The Kaplan–Meier curves. **A** Local tumor progression-free survival. **B** Overall survival

The cumulative 1- and 3-year LTPFS rate was 54.1% and 40.5% in the brachytherapy group versus 58.3% and 41.7% in the MWA group (*P* = 0.524 and *P* = 0.889 for group comparison at 1 and 3 years, respectively; Table 2). The median LTPFS was 17.3 months (95% confidence interval [CI]: 8.4–19.2) in the brachytherapy group versus 19.8 months (95% CI: 8.3–21.9) in the MWA group (*P* = 0.871), respectively (Fig. 5A). In multivariable Cox regression, longer LTPFS was associated with lesion size ≤ 3 cm (HR = 1.437; 95% CI: 0.893–3.594) and serum CEA < 10 ng/ml (HR = 2.346; 95% CI: 1.240–4.436) (Table 3). LTPFS did not differ significantly between the patients with 5–10 versus > 10 mm MAM in the MWA group (Table 4).

**Table 3:** Cox regression analysis of the entire cohort

	Univariable Cox regression		Multivariable Cox regression	
	HR (95% CI)	P	HR (95% CI)	P
**LTPFS** Sex				
Female				
Male	0.359 (0.153–1.098)	0.146		
Age (years)				
<65				
≥65	0.684 (0.327–1.753)	0.618		
Treatment of lung metastasis				
L-SABT				
MWA	0.349 (0.103–0.568)	0.083		
Tumor diameter				
≤3 cm				
>3 cm	0.925 (0.258–3.094)	0.025	1.437 (0.893–3.594)	0.021
Distribution of lung metastasis				
Unilateral				
Bilateral	2.357 (0.961–5.336)	0.257		
Distance from hilum pleura/interlobar fissure				
≤10 mm				
>10 mm	3.453 (1.568–8.752)	0.056	3.132 (1.083–6.547)	0.097
Lung metastasis				
Metachronous				
Synchronous	3.104 (1.476–5.331)	0.037	1.673 (0.937–3.961)	0.298
CEA(ng/ml)				
<10				
≥ 10	2.802 (1.531–5.126)	0.001	2.346 (1.240–4.436)	0.017
**OS**				
Sex Female Male	0.356 (0.189–1.235)	0.097	0.641 (0.235–1.672)	0.253
Age (years) < 65 ≥ 65	0.757 (0.368–1.696)	0.492		
Treatment of lung metastasis				
L-SABT				
MWA	0.472 (0.225–1.428)	0.163		
Tumor diameter				
≤3 cm				
>3 cm	1.576 (0.446–4.684)	0.095	1.987 (1.068–3.722)	0.036
Distribution of lung metastasis				
Unilateral				
Bilateral	1.779 (0.432–4.775)	0.229		
Distance from hilum/pleura/interlobar fissure				
≤10 mm				
>10 mm	2.984 (1.097–6.403)	0.087	2.531 (0.924–5.773)	0.151
Lung metastasis				
Metachronous				
Synchronous	2.577 (1.458–4.669)	0.062	1.332 (0.859–3.452)	0.338
CEA (ng/ml)				
<10				
≥10	2.996 (1.345–5.667)	0.004	2.698 (1.042–4.337)	0.013

LTPFS, local tumor progression-free survival; HR, hazard ratio; CI, confidence interval; L-SABT, low-dose rate stereotactic ablative brachytherapy; MWA, microwave ablation; CEA, carcinoembryonic antigen; OS, overall survival.

**Table 4:** Outcome analysis stratified by MAM in the MWA group

Characteristics	MAM 5–10 mm (*N* = 26)	MAM > 10 mm (*N* = 22)	P value
LTPFS at 1 year, no. (%) Yes No	14 (53.8%) 12 (46.2%)	12 (54.5%) 10 (45.5%)	> 0.99
LTPFS at 3 years, no. (%) Yes No Median LTPFS (months) OS at 1 year, no. (%) Yes No OS at 3 years, no. (%) Yes No	10 (38.5%) 16 (61.5%) 18.4 (95% CI: 8.7–20.6) 18 (69.2%) 8 (30.8%) 12 (46.2%) 14 (53.8%)	10 (45.5%) 12 (54.5%) 19.3 (95% CI: 8.6–21.7) 16 (72.7%) 6 (27.3%) 12 (54.5%) 10 (45.5%)	> 0.99 0.965 > 0.99 > 0.99
Median OS (months) Complications Pneumothorax Bronchopleural fistula Pleural effusion Hydropneumothorax Fever Total, no. (%)	22.5 (95% CI: 16.1–33.7) 8 (30.8%) 0 2 (7.7%) 0 4 (15.4%) 14 (53.8%)	23.2 (95% CI: 15.8–35.6) 8 (36.4%) 6 (27.3%) 0 2 (9.1%) 2 (9.1%) 18 (81.8%)	0.897 0.211

MWA, microwave ablation; MAM, minimal ablation margin; LTPFS, local tumor progression-free survival; OS, overall survival.

**Table 5:** Subgroup analysis stratified by distance from key structures

Characteristics	Brachytherapy group (*N* = 74)	MWA group (*N* = 48)	P value
**≤ 10 mm from hilum/pleura/interlobar fissure** Demographics and baseline Sex, no. (%) Male Female	*N* = 54 34 (63%) 20 (37%)	*N* = 18 10 (55.6%) 8 (44.4%)	0.712
Mean age (years) Age (years), no. (%) <65 ≥65 Primary tumor location, no. (%) Right colon Left colon Rectum Maximum tumor diameter (cm, mean ± SD) Tumor size, no. (%) ≤3 cm >3 cm Tumor number, no. (%) 1 2 3 Distribution of lung metastasis, no. (%) Unilateral Bilateral	65.9 ± 6.7 32 (59.3%) 22 (40.7%) 24 (44.4%) 16 (29.6%) 14 (26) 3.9 ± 1.3 30 (51.4%) 24 (48.6%) 12 (22.2%) 26 (48.2%) 16 (29.6%) 34 (63%) 20 (37%)	61.5 ± 8.2 12 (66.7%) 6 (33.3%) 6 (33.3%) 6 (33.3%) 6 (33.3%) 2.8 ± 0.7 12 (66.7%) 6 (33.3%) 8 (44.5%) 6 (33.3%) 4 (22.2%) 10 (55.6%) 8 (44.4%)	0.263 > 0.99 0.892 0.067 0.439 0.612 0.712
Location of lung metastasis, no. (%) Adjacent to the hilum Adjacent to the pleura/interlobar fissure Lung metastasis, no. (%) Metachronous Synchronous CEA (ng/ml), no. (%) <10 ≥10 Complication, no. (%). Pneumothorax Bronchopleural fistula Pleural effusion Hydropneumothorax Survival LTPFS at 1 year, no. (%) Yes No LTPFS at 3 years, no. (%) Yes No Median LTPFS (months) OS at 1 year, no. (%) Yes No	28 (51.9%) 26 (48.1%) 38 (70.4%) 16 (29.6%) 18 (33.3%) 36 (66.7%) 16 (29.6%) 10 (18.5%) 0 4 (7.4%) 2 (3.7%) 30 (55.6%) 24 (44.4%) 20 (37%) 34 (63%) 17.4 (95% CI: 8.9–23.1) 40 (74.1%) 14 (25.9%)	6 (33.3%) 12 (66.7%) 8 (44.4%) 10 (55.6%) 8 (44.4%) 10 (55.6%) 16 (88.9%) 8 (44.4%) 6 (33.3%) 0 2 (11.1%) 8 (44.4%) 10 (55.6%) 6 (33.3%) 12 (66.7%) 16.8 (95% CI: 8.4–22.7) 12 (66.7%) 9 (33.3%)	0.451 0.235 0.693 0.005 0.706 > 0.99 0.793 0.686
OS at 3 years, no. (%) Yes No Median OS (months) **> 10 mm from hilum/pleura/interlobar fissure** Demographics and baseline Sex, no. (%) Female Male Mean age (years) Age (years), no. (%) < 65 ≥ 65 Primary tumor location, no. (%) Right colon Left colon Rectum Maximum tumor diameter (cm, mean ± SD) Tumor number, no. (%) 1 2 3 Distribution of lung metastasis, no. (%) Unilateral Bilateral Lung metastasis, no. (%) Metachronous Synchronous CEA (ng/ml), no. (%) < 10 ≥ 10 Complication, no. (%) Pneumothorax Pleural effusion Hydropneumothorax Fever Survival LTPFS at 1 year, no. (%) Yes No LTPFS at 3 years, no. (%) Yes No Median LTPFS (months) OS at 1 year, no. (%) Yes No OS at 3 years, no. (%) Yes No Median OS (months)	26 (48.1%) 28 (51.9%) 22.5 (95% CI: 15.6–32.1) *N* = 20 8 (40%) 12 (60%) 61.3 ± 6.5 14 (70%) 6 (30%) 6 (30%) 4 (20%) 10 (50%) 7.1 ± 1.5 6 (30%) 8 (40%) 6 (30%) 10 (50%) 10 (50%) 18 (90%) 2 (10%) 10 (50%) 10 (50%) 18 (90%) 10 (50%) 2 (10%) 6 (30%) 0 10 (50%) 10 (50%) 10 (50%) 10 (50%) 16.9 (95% CI: 7.8–23.6) 16 (80%) 4 (20%) 10 (50%) 10 (50%) 19.3 (95% CI: 14.9–34.6)	8 (44.4%) 10 (55.6%) 20.8 (95% CI: 16.1–33.6) *N* = 30 16 (53.3%) 14 (46.7%) 56.5 ± 4.8 16 (53.3%) 14 (46.7%) 12 (40%) 10 (33.3%) 8 (26.7%) 2.1 ± 0.5 10 (33.3%) 8 (26.7%) 12 (40%) 14 (46.7%) 16 (53.3%) 26 (86.7%) 4 (13.3%) 16 (53.3%) 14 (46.7%) 16 (53.3%) 8 (26.7%) 2 (6.7%) 0 6 (20%) 20 (66.7%) 10 (33.3%) 14 (46.7%) 16 (53.3%) 20.1 (95% CI: 8.6–23.8) 12 (40%) 18 (60%) 16 (53.3%) 14 (46.7%) 25.3 (95% CI: 14.7–38.3)	> 0.99 0.65 0.688 0.153 0.678 0.596 0.036 0.879 > 0.99 > 0.99 > 0.99 0.088 0.442 > 0.99 0.671 0.099 > 0.99 0.787

MWA, microwave ablation; CEA, carcinoembryonic antigen; LTPFS, local tumor progression-free survival; OS, overall survival.

### OS

The cumulative 1- and 3-year OS rate was 75.7% and 48.6% in the brachytherapy group versus 75% and 50% in the MWA group (*P* = 0.775 and 0.918 for group comparison at 1 and 3 years, respectively; Table 2). The median OS was 21.6 months (95% CI: 15.3–33.9) in the brachytherapy group versus 23.4 months (95% CI: 15.4–36.7) in the MWA group (*P* = 0.865, Fig. 5B). In the multivariable regression, longer OS was associated with lesion size ≤ 3 cm (HR = 1.987; 95% CI: 1.068–3.722) and serum CEA < 10 ng/ml (HR = 2.698; 95% CI: 1.042–4.337) (Table 3). OS did not differ significantly between the patients with 5–10 versus > 10 mm MAM in the MWA group (Table 4).

### Complications

The two groups did not differ in the rate of overall complications (45.9% in the brachytherapy group versus 66.7% in the MWA group, *P* = 0.113; Table 2). No procedure-related death or grade 4 complications occurred in either group. The rate of grade 2–3 complications was 5.4% (4/74) in the brachytherapy group (two case each for pneumothorax and hydropneumothorax requiring drainage) versus 16.7% (8/48) in the MWA group (two cases for pneumothorax and six cases for bronchopleural fistula requiring drainage) (*P* = 0.2). Grade 1 complications included pneumothorax, pleural effusion, hydropneumothorax and fever, and did not differ between the two groups. Seed migration into the thoracic cavity occurred in two patients (2.7%) during the follow-up, but no radiation-induced pleuritis was observed.

In the subgroup analysis that included only patients with lesions within 10 mm from the hilum, pleura or interlobar fissure, the rate of complications was 29.6% (16/54) in the brachytherapy group and 88.9% (16/18) in the MWA group (*P* = 0.005; Table 5). Specific complications included pneumothorax (10/54 in the brachytherapy group versus 8/18 in the MWA group), bronchopleural fistula (none in the brachytherapy group versus 6/18 in the MWA group), pleural effusion (4/54 in the brachytherapy group versus none in the MWA group), and hydropneumothorax (two each in the two groups).

## Discussion

The European Society for Medical Oncology [22] stipulated that the best treatment for oligometastasis should be selected based on comprehensive evaluation of all available information, including the size and localization of the metastases, reported local control rate, and invasiveness. Surgical resection remains the standard treatment for lung metastasis, but only 25–30% of patients are appropriate candidates due to old age, low cardiopulmonary reserve and prevalent comorbidities [23]. In selected cases, local treatments could prolong survival in addition to symptom alleviation [24–27]. Among the options of local treatments, the efficacy of L-SABT and MWA have been well established [10, 28].

In the current study, L-SABT and MWA were comparable in LTPFS and OS rates. Also, LTPFS and OS did not differ significantly in the patients with 5–10 versus > 10 mm MAM in the MWA group. The rate of complications was also similar between the two groups. However, in the subgroup analysis that only included patients with lesions within 10 mm from the hilum, pleura or interlobar fissure, the rate of complications was substantially lower in the brachytherapy group, primarily driven by lower rate of bronchopleural fistula.

Kurilova et al. reported 1- and 3-year LTPFS were 93% and 86% in a study of MWA for CRC lung metastasis [14]. The 1- and 3-year LTPFS were (58.3% and 41.7%) in the MWA group in the current study was apparently lower than that reported by Kurilova et al., likely due to larger tumor diameter in the current study (2.5 ± 0.8 cm versus 1 cm in the Kurilova study).

Compared with radiofrequency ablation, MWA could achieve a larger and more uniform ablation zone [16, 29]. In a study of patients with CRC lung metastases, Vogl and colleagues reported a higher rate of local control (88.3%) with MWA than radiofrequency ablation (69.2%) [30]. A major disadvantage of MWA is the limited output power; as such, MWA is usually recommended only for lesions with < 3 cm diameter. The maximum tumor diameter in the MWA group of the current study (2.5 ± 0.8 cm) is consistent with such a pattern.

The maximum tumor diameter in the L-SABT group in the current study was significantly higher (5.7 ± 2.4 cm) than in the MWA group. Despite of such a difference, LTPFS and OS were similar in the two groups. A notable finding in the current study was the much higher rate of bronchopleural fistula in the MWA group versus in the brachytherapy group in the subgroup analysis that only included patients with lesions within 10 mm from the hilum, pleura or interlobar fissure. These findings are consistent with the susceptibility of nearby structures to the thermal injury by MWA [31]. A previous study by Vogl and colleagues reported a lower rate of local tumor control in tumors located < 5 cm versus >5 cm from the hilum, likely due to the safety concern and thus lower energy for tumors closer to the hilum [30].

Iodine-125 seeds exhibit several unique properties, including a short half-valence layer [32, 33]. Accordingly, L-SABT often requires several puncture needles and dozens of seeds [34]. Longer procedural time with L-SABT in the current study is consistent with such a characteristic. Another major limitation of brachytherapy is the impact on the social activity of the patients.

Similar to previous studies [26, 35, 36], the multivariable Cox regression in the current study showed that improved prognosis was associated with smaller lesion size and lower CEA level, thus supporting the validity of the key findings.

The current study has several key limitations. First, the study was retrospective in nature, and thus subjected to major selection bias. Having said this, such biases (e.g., higher maximum lesion size in the brachytherapy group) favors the MWA group in terms of survival outcomes. Despite of such a bias, local tumor control, LTPFS and OS were similar between the two groups, adding support to the utility of brachytherapy. Second, the sample size was fairly small. Prospective studies with larger sample size are needed to verify our preliminary findings.

## Conclusions

L-SABT and MWA were both effective for CRC oligometastasis to the lungs. Considering the caveats associated with radioisotope use in L-SABT, MWA is generally preferable in patients with lesion > 10 mm from the key pulmonary structures. In patients with lesion within 10 mm from the key pulmonary structures, however, L-SABT could be considered as an alternative due to lower risk of bronchopleural fistula.

## Data Availability

The datasets supporting the conclusions of this article are available from the corresponding author on reasonable requests.
